# Assessing the In Vitro Individual and Combined Effect of *Arthrobotrys oligospora* and *A. musiformis* (Orbiliales) Liquid Culture Filtrates against Infective Larvae of the Sheep Blood-Feeding Nematode *Haemonchus contortus* (Trichostrongylidae)

**DOI:** 10.3390/pathogens13060498

**Published:** 2024-06-11

**Authors:** Antonio Colinas-Picazo, Pedro Mendoza-de Gives, Gustavo Pérez-Anzúrez, Enrique Gutiérrez-Medina, Génesis Andrea Bautista-García, Edgar Jesús Delgado-Núñez, Agustín Olmedo-Juárez

**Affiliations:** 1 Laboratory of Helminthology, National Centre for Disciplinary Research in Animal Health and Innocuity (CENID-SAI), National Institute for Research in Forestry, Agriculture and Livestock, INIFAP-SADER, Morelos, Jiutepec 62550, Mexico; antoniocolinasp@gmail.com (A.C.-P.); tavopzaz@gmail.com (G.P.-A.); egm.20797@gmail.com (E.G.-M.); bagg150583@gmail.com (G.A.B.-G.); aolmedoj@gmail.com (A.O.-J.); 2 Faculty of Agricultural, Livestock and Environmental Sciences, Autonomous University of the State of Guerrero, Iguala de la Independencia 40040, Mexico; edgarjezuz@gmail.com

**Keywords:** nematophagous fungi, *Arthrobotrys*, *Haemonchus*, predation, myco-compounds, nematocidal activity

## Abstract

Background: *Arthrobotrys* species are nematophagous fungi that secrete extracellular nematocidal products (ECP). The individual and combined effects of ECP from *Arthrobotrys oligospora* (*Ao*) and *A. musiformis* (*Am*) growth in liquid media against *Haemonchus contortus* L3 (HcL3) were assessed. Methods: The isolation, morphological (MI) and molecular identification (Mol-I), assessment of nematocidal activity (NA) of fungal liquid culture filtrates (LCF) in two liquid media alone and in combination and the myco-compound profile identification (MCP) were performed. Results: The MI suggested that the fungi corresponded to the species *Ao* and *Am*. This result was confirmed by PCR analysis followed by sequencing, alignment and a phylogenetic analysis. Likewise, the highest *Hc* mortalities were 91.4% with individual LCF of *Am* and 86.2% with those of *Ao* at the highest concentration (100 mg/mL) in Czapek-Dox Broth. The combination of both LCF resulted in a similarly high larval mortality with no statistical differences in relation to individual activity (*p* > 0.05). The MCP showed the presence of alkaloids in both fungi. Coumarins, sterols and saponins were found only in *Ao*. Main conclusions: Both fungi produced ECP with a high NA that could be identified and assessed in future studies as potential natural anthelmintic compounds.

## 1. Introduction

*Haemonchus contortus* is a blood-feeding parasitic nematode living in the abomasum (stomach) of small ruminants, that provokes gastritis, blood-loss in the abomasum, anorexia, anaemia, lethargy, weakness, weight loss, emaciation and, in severe cases, the death of young animals [1,2]. This and other genera/species of parasitic nematodes live in the gastrointestinal tract of small ruminants, where they cause severe deterioration of flock health and productivity. Gastrointestinal nematodiasis is controlled by the frequent and continuous administration of chemical anthelmintic drugs to the animals; however, the development of anthelmintic resistance in parasites has led to increasing inefficacy of these drugs, raising alarm among farmers worldwide [3,4]. During recent decades, strategies other than the use of chemical anthelmintic drugs have been explored, including the use of natural antagonists of nematodes, such as a group of micro-fungi called nematode-trapping fungi [5,6]. Nematode-trapping fungi are regular microorganisms from the soil mycobiota living as saprophyte organisms and taking their nitrogen and carbon sources from decaying wood and litter; however, they can transform their saprophyte lifestyle into a predatory or parasitic one in the presence of nematodes such that, once trapped, they are used as a food source by fungi [7]. The species *Arthrobotrys oligospora* and *A. musiformis* have been classified as Orbiliales fungi [8]. These species develop three-dimensional adhesive nets from their mycelia where nematodes are trapped and destroyed for eventual use by fungi as their main source of nutrients [9]. In addition to the mechanical capture exerted by trapping devices, nematode-trapping fungi possess other strategies to kill and penetrate nematodes using myco-chemical constituents, including enzymes and products derived from secondary metabolism [10]. The objectives of the present study were to isolate nematode-trapping fungi and perform a taxonomical identification via morphological and molecular procedures, as well as to assess the nematocidal activity of their liquid culture filtrates, either individually or combined, and produced in two liquid media (sweet potato dextrose broth [SPDB] and Czapek-Dox Broth [CzDB]) (Sigma, Aldrich, Merck, Darmstadt, Germany) against *H. contortus* infective larvae, and eventually to identify associated groups of myco-compounds.

## 2. Materials and Methods

### 2.1. Nematodes

#### 2.1.1. Obtaining the Free-Living Nematode *Panagrellus redivivus* for Use as Bait to Isolate Nematophagous Fungi

A population of the free-living nematode *P. redivivus* was provided as fish food by a local pet store in Jiutepec Municipality, Morelos, Mexico. For *en masse* reproduction, nematodes were cultured in sterile plastic bowls containing sterile oat flakes (20 g), and 200 mL of sterile distilled water was added. Oat flakes and water were mixed and homogenized to finally obtain a humid mass that was used as a nutrient substrate for bacteria, and in turn, bacteria were used as the main source of food by the free-living nematodes. Bowls were covered with a cap of foil and gauze to prevent mosquitoes from entering. Cultures were incubated at room temperature (18–28 °C) for 7 days [11]. After the incubation period, some culture material was collected using a metal spoon and dissolved in a glass of water, producing many *P. redivivus* specimens swimming in an aqueous suspension. Nematodes were separated from the culture medium through a 74-µm sieve. This step was repeated several times until the nematodes were very clean. Nematodes were resuspended in sterile distilled water and sieved through a coffee filter. Nematodes were eventually recovered using the Baermann funnel technique [12].

#### 2.1.2. Procurement of *Haemonchus contortus* Infective Larvae (L_3_)

A 4-month-old pelibuey lamb was used. The McMaster technique using the faecal matter of this lamb was negative for the presence of gastrointestinal nematode eggs. This lamb was orally inoculated with 350 *H. contortus* infecting larvae per kilogram of body weight. After a pre-patent period of 21 days, faecal samples taken directly from the rectum of this lamb tested positive for the presence of nematode eggs by the McMaster technique. All rules regarding the treatment of animals and the prevention of unnecessary animal suffering were carefully followed according to the Norma Oficial Mexicana (Official Mexican Standard) with official rule number NOM-052-ZOO-1995 (http://www.senasica.gob.mx, accessed on 8 August 2023). Additionally, the Ley Federal de Sanidad Animal (Federal Law for Animal Health) DOF 07-06-2012 was strictly followed in accordance with the ethical standards outlined by INIFAP. Fresh faeces were ground in a plastic bowl and mixed with small pieces of polyurethane foam to obtain a porous mass that retained the oxygen necessary for the optimum development of nematode eggs [13]. Faecal cultures were incubated at room temperature (18–28 °C) for 7 days. After 5–7 days of elaboration of faecal cultures, a rather large amount of *H. contortus* infective larvae was collected using the Baermann Funnel technique for 24 h [13,14]. Infective larvae of the parasite were cleaned using the differential centrifugation technique with 40% sucrose density gradients [15]. After centrifugation for 5 min at 3500 rpm, a white ring in the interphase between water and sucrose corresponding to clean larvae was visualized. Larvae were removed with a Pasteur pipette and deposited into assay tubes, which were filled with water. In order to discard sucrose residue from the aqueous suspension of larvae, the assay tubes containing larvae in suspension were centrifuged at the same speed and spin times, and larvae were sedimented to the base of the assay tubes. The supernatant was discarded, and the tubes were refilled with sterile water and centrifuged again. After three to four centrifugations, larvae in the sediment were free of sucrose residues.

### 2.2. Isolation of Nematophagous Fungi

Two 50-g individual samples of soil from a poultry farm in Cuernavaca Municipality, state of Morelos, Mexico were taken and transported in plastic bags to the Laboratory of Helminthology of CENID-SAI in Jiutepec, Morelos. A small soil sample (approximately 0.5 g) was sprinkled on three sterile water agar plates. Plates were incubated at room temperature (18–28 °C) for three days. After this period, some drops of an aqueous suspension containing an undetermined number of specimens of the free-living nematode *P. redivivus* were added to each plate to promote the growth of nematophagous fungi. After one week, the agar surface was observed under the microscope, and aerial structures typical of nematophagous fungi were seen, including trapping devices, conidiophores and trapped nematodes. These structures were transferred to fresh sterile water agar plates using a sterile metal needle. Plates were maintained at the same temperature, and aerial structures were again transferred to sterile water agar plates. This process was repeated until fungi were eventually obtained in pure culture [16]. 

### 2.3. Procurement of Fungal Liquid Culture Filtrates

Fungi were cultivated in two different liquid culture media: SPDB and CzDB. Briefly, SPDB was prepared using 200 g of organic sweet potato (obtained from an organic farm at the Autonomous University of the State of Morelos (UAEM) in Cuernavaca, city). Sweet potato was peeled and chopped into small, square pieces of approximately 1 cm^2^. Pieces of sweet potato were cooked in 1 L of distilled water and boiled for 25 min. Subsequently, cooked medium was sieved using a piece of gauze to separate the solid material, and 20 g of dextrose was added to the liquid phase and the volume adjusted to 1 L with distilled water. The liquid was transferred to 250 mL flasks, depositing 50 mL of media in each flask (n = 3). Flasks containing the medium were sterilized in an autoclave and allowed to cool. Fifty hundred microliters of penicillin (50 I.U./mL)/streptomycin (50 μg/mL) were added to each flask. This procedure was performed using a laminar flue cabinet. Later, three plugs (1 cm^2^) obtained from the surface of a water agar plate containing a 21-day-old fungal culture were deposited in each flask. This procedure was performed for each of the two fungi. The same number of flasks with only liquid medium and without any fungal inoculum was used as a negative control. After the incubation period, liquid culture filtrates were obtained as follows: mycelia were separated by filtration using different kinds of filters and pore diameters, including a Whatman^®^ #4 25 µm filter paper (General Electric Company, Boston, MA, USA), and passed through three Millipore^®^ filters (2 µm, 0.45 µm and 0.22 µm). Filtration was achieved using a Millipore filtration unit connected to a vacuum pump (Millipore High Performance Pump115 V/60 Hz, Darmstadt, Germany). Filtered media were concentrated using a rotatory evaporator (Büchi^®^ R-300, Zürich, Switzerland) to eliminate the greatest volume of water without altering the sample characteristics. Finally, evaporated material was lyophilised using a LABCONCO^®^ FreeZone 4.5 plus, U.S.A lyophiliser (Labconco, Kansas, MO, USA). 

### 2.4. Traditional Taxonomic Identification of Fungal Isolates by Morphometry

The morphological identification of fungi was performed by both macroscopic and microscopic observations. In the case of macroscopic characteristics of both fungi growing in water-agar and potato dextrose agar were described. Regarding the microscopic characteristics of fungi, aerial structures such as conidia, conidiophores, trapping devices and the presence or absence of chlamydospores, among other characteristics, were observed and analysed under the microscope (20× and 40× objectives). Twenty-five conidia and conidiophores were randomly taken, and their dimensions, i.e., height and width, were measured and recorded. Taxonomic morphometric identification was achieved by comparing the characteristics of our isolates with those described in specialized taxonomic keys [17,18,19]. Additionally, a set of microphotographs of the aerial structures of taxonomical importance were taken in a Leica DM6 B optical microscope (Leica, Wetzlar, Germany).

### 2.5. Molecular Identification

Fungi were recovered from CzDB cultures and processed to obtain DNA using the Wizard^®^ Genomic DNA Purification Kit (PROMEGA, Madison, WI, USA). Genomic material was quantified using an IMPLEN (NanoPhotometer NP80) spectrophotometer. The endpoint PCR technique was performed following the procedures standardized at the Laboratory of Helminthology of CENID-SAI, INIFAP according to the methodology described by Tigano-Milani et al. (1995) [20]. The Internal Transcribed Spacer (ITS)-1, ITS-2 and 5.8S complete regions and a partial sequence of small 18S and large 28S sub-unit regions were amplified. The selected primers were ITS5-forward (5′-TCC TCC GCT TAT TGA TAT GC-3′) and ITS4-reverse (5′-GGA AGT AAA AGT CGT AAC AAG G-3′) [21].

The PCR technique is briefly described next. The reaction was carried out in a 20-µL total volume, containing 100 ng of gDNA, 10 µL GoTaq^®^ Green Master Mix 2X (PROMEGA, Madison, WI, USA), 1.5 µL of each primer at 20 µM and nuclease-free water to a total volume of 20 µL. The PCR method was carried out using a C1000 Touch^®^ Thermal Cycler (BIORAD, Hercules, CA, USA). The PCR conditions were established as follows: initial denaturation at 94 °C for 3 min; an amplification stage, including 35 cycles of denaturation at 94 °C for 1 min, annealing at 42 ºC for 90 s, extension at 72 °C for 90 s; and a final extension stage at 72 °C for 5 min. 

The Wizard^®^ SV Gel and PCR Clean-Up System (Promega, Madison, WI, USA) was used to purify the PCR products. Genomic material was sequenced at the Institute of Biotechnology of the National Autonomous University (IBT-UNAM), Cuernavaca city, Morelos, Mexico, using an Applied Biosystem Sequencer (7700; Thermo Fisher Scientific, Waltham, MA, USA). Similarity and coverage of sequences were achieved using the BLAST tool from NCBI. The sequences were compared with the closest sequences previously reported in the NCBI database. Molecular analysis was used to confirm the morphological identification [22]. Multiple alignment with our sequences and 47 related species from *Arthrobotrys*, *Dactylellina* and *Drechslerella* genera was performed using MEGA software (v11.0.13). The best substitution model was estimated by JModelTest software (v2.1.10). Maximum likelihood (ML) analysis was made in IQTree (v1.6.12) to construct the best phylogenetic tree; bootstrap support values (BS) were obtained by ultrafast bootstrapping analysis employing 10,000 replicates. FigTree software (v1.3.1) was used to visualize and edit the generated tree.

### 2.6. Assessing the Predatory Activity of Fungi against Haemonchus contortus Infective Larvae

Thirty water agar plates (60 mm diameter) were used. Plates were divided into three groups (n = 10) and named as groups 1–3. Plates of groups 1 and 2 were added with a square piece of water agar (1 cm^2^) from 10-day-old culture plates of *A. oligospora* and *A. musiformis*; respectively. Plates of group 3 contained only water agar to be used as the control group. Plates containing fungi were incubated for 10 days at room temperature (18–28 °C). After the incubation period, 100 µL of PBS (7.2 pH) containing two hundred *H. contortus* infective larvae were added to the surface of each plate of the three experimental groups. All plates were incubated for 10 days at the same temperature mentioned above. After incubation, the agar from every plate was individually transferred to a Baermann funnel apparatus where it remained for 24 h. The face of the agar on which nematodes and fungi were located was placed toward the bottom of the assay tubes to allow non-trapped larvae to freely migrate and eventually remain sedimented at the bottom of the tubes. Non-trapped larvae from nematode/fungus interactions and whole larvae from control groups were recovered and quantified. The number of larvae in ten 5-µL aliquots from a 3-mL total volume recovered from experimental plates was quantified under a microscope (5×), and the mean numbers of larvae per group were estimated. The whole experiment was performed in triplicate. 

The larval reduction percentage attributed to the predatory effect exerted by fungi was estimated using Abbott’s formula:

(1) $$Larval\;Reduction\;\%=\frac{\left(RLCgroup-RLinteraction\right)}{RLCgroup}100$$

where:

RLCgroup = Recovered larvae from the control group

RLinteraction = Recovered larvae from the fungi/larvae interaction group

### 2.7. Assessing the Nematocidal Activity of Fungal Liquid Culture Filtrates against Haemonchus contortus Infective Larvae 

**Figure 1:**
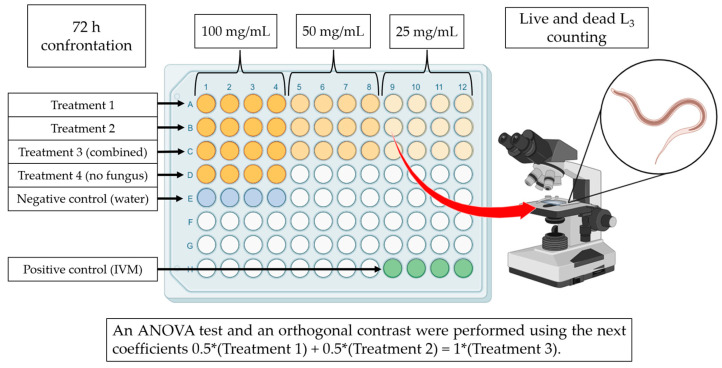
Scheme representing the steps of the experimental process to evaluate the lethal effect of liquid culture filtrates obtained with two nematophagous fungi *Arthrobotrys oligospora* and *A. musiformis* growth in two culture media, Sweet Potato Dextrose Broth and Czapek-Dox Broth, against *Haemonchus contortus* infective larvae.

The interaction between Liquid Culture Filtrates (LCF) and *H. contortus* infective larvae was carried out using 96-well microtiter plates (n = 4). The dried LCF were reconstituted using Phosphate Buffered Saline (PBS). Ten experimental groups were established to assess the lethal effect of LCF obtained from *A. oligospora* and *A. musiformis* growing in two media SPDB and CzDB. LCF of both fungi were assessed individually and combined (50:50) at three different concentrations (25, 50 and 100 mg/mL). Proper control with only the media was used to discard any possible lethal effect of these media on the nematodes. Additionally, PBS and ivermectin 0.5% were also used as negative and positive controls (Table 1). Fifty microliters of the corresponding treatment and 50 µL of an aqueous larval suspension (in PBS) containing approximately 100 *H. contortus* infective larvae were deposited in every one of the four wells per treatment. All plates were incubated at room temperature (18–28 °C) for 72 h (Figure 1). The whole experiment was performed in triplicate.

After incubation, the volume of each well was observed under the microscope, and dead and live larvae were counted following the previously described procedure. Motionless larvae were determined to be alive or dead by applying a physical stimulus by touching its cuticle using a sharp metallic needle. Live larvae normally start to move actively after this stimulus. Larvae that remain motionless after this stimulus are considered dead larvae [23]. The whole experiment was repeated three times. Larval mortality obtained for each treatment and control group was calculated. The larval mortality attributed to the effect of LCF of each fungus and each concentration was estimated using the following formula:

(2) $$\%\;Larval\;Mortality=\frac{\left(Mortality\;L3\;Treated\;group-Mortality\;L3\;Control\;group\right)}{\left(1-Mortality\;L3\;Control\;group\right)}100$$

where:

Mean L3 Treated group = Mean of larval mortality from treated group.

Mean L3 Control group = Mean of larval mortality from control group. 

### 2.8. Microscopic Analysis of Haemonchus contortus Infective Larvae Exposed to Fungal Liquid Culture Filtrates

After exposure of *H. contortus* larvae to LCF, a random selection of larvae from the different treatments was carried out to photograph possible morphological changes attributed to the effect of compounds present in the fungal filtrates. Larvae were photographed using a LEICA DM6 compound microscope using the program LAS V4.9 to document our findings.

### 2.9. Myco-Chemical Profile

A myco-quantitative reagent analysis was carried out using standard procedures with the proper reagents and methods. Alkaloids were determined using Dragendorff Mayer and Wagner’s reagents, and the Bornträger test was performed to identify the presence of coumarins. Likewise, the presence of flavonoids was investigated using Mg^2+^ and HCl tests. On the other hand, the ferric chloride, gelatine and saline solution tests were carried out to identify tannins. Finally, the Lieberman–Burchard and Salkowski tests were used to identify the presence of Triterpenes [24].

### 2.10. Statistical Analysis

Data obtained from the predatory activity assay of both fungal isolates were individually analysed by the Student’s *t*-test, where the larval reduction percentage was obtained by comparing the mean number of recovered larvae from the nematode/fungi interaction plates and from the control group without fungus. For the results of the nematocidal activity of LCF, a completely random model was used, and ANOVA was performed where the means of larval mortality in the different treatments was considered as the dependent variable. An orthogonal contrast by the Bonferroni method was performed to compare the effect of the combination of both LCF from fungi grown in the same media using the following coefficients: 0.5(AspT, fungus 1) + 0.5(AspG, fungus 2) − 1(combined, 50:50). A *p* value of 0.05 was used for all tests.

## 3. Results and Discussion

### 3.1. Traditional Taxonomy (Morphometrics)

Two isolates of nematode-trapping fungi were obtained, and an institutional collection code name was designed as AspT and AspG isolates. 

**Figure 2:**
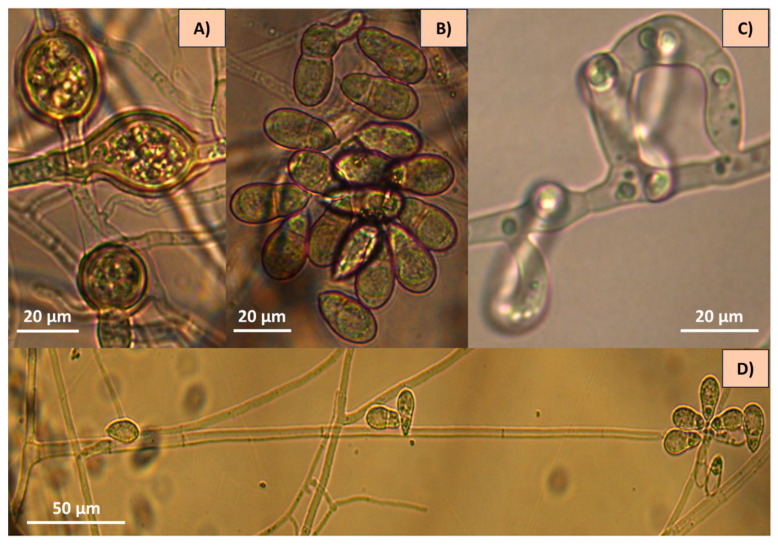
Microphotographs of *Arthrobotrys oligospora* showing structures of taxonomic importance: (**A**) chlamydospores, (**B**) conidia, (**C**) three-dimensional adhesive nets and (**D**) conidiophore.

After the main structures of taxonomic interest were observed and analysed under the microscope (including conidia and conidiophore measurements), the morphological characteristics were compared with those described in taxonomic keys, and the authors eventually decided to classify these fungi as *A. oligospora* and *A. musiformis.* The isolate AspT showed hyphae, conidia and hyaline conidiophores with repeated proliferation of conidia. Conidia showed a globose shape, lightly constricted in their septum. This fungus showed the presence of three-dimensional adhesive nets (Figure 2).

**Figure 3:**
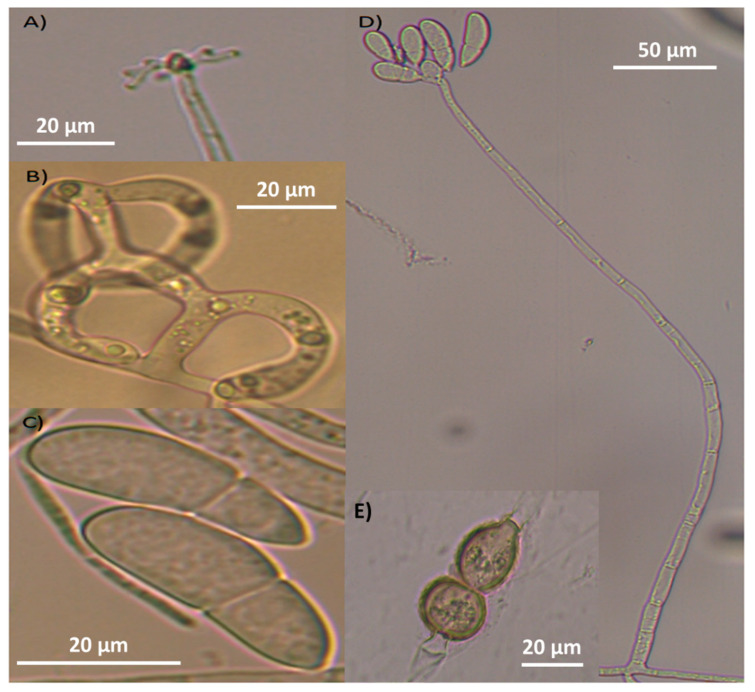
*Arthrobotrys musiformis* structures of taxonomic importance: (**A**) Candelabrum; (**B**) Three-Dimensional adhesive net; (**C**) Conidia; (**D**) Conidiophore and (**E**) Chlamydospores.

Likewise, the analysis of the isolate AspG showed hyaline hyphae, conidia and conidiophores. Nevertheless, conidiophores showed long and cylindrical denticles, produced by candelabra, where conidia were generated. Conidia were elongated and slightly curved, typical of *A. musiformis*. This isolate showed the same trapping devices corresponding to the three-dimensional adhesive nets described above (Figure 3).

The summarized information about measurements and observations of the main morphological characteristics of both isolates are shown in Table 2.

At first sight, the growth of erect and long-stem apical conidiophores crowned by conidia clusters of globose form, as well as the presence of one septum almost at the middle of the conidia in the AspT strain suggested to us the presence of a nematophagous fungus belonging to the genus *Arthrobotrys* [18]. However, there are several species sharing these characteristics, and this fact can confuse the taxonomic identification. Some of the species sharing these similar characteristics are *A. oligospora*, *A. robusta*, *A. superba*, *A. conoides* and *A. arthrobotryoides*, among others. After observing the characteristics in more detail, including the measurements of some structures, i.e., conidia length and width, the number of conidia in the clusters, the presence or absence of branched conidiophores, the presence or absence of chlamydospores and particularly, the type of trapping devices, we were able to differentiate our isolate from other species. Because our isolate showed only unbranched conidiophores, and the measurements of conidia and the length of conidiophores were similar to those of species described in the taxonomical identification keys, we were able to discard several species that do not share these characteristics. 

**Figure 4:**
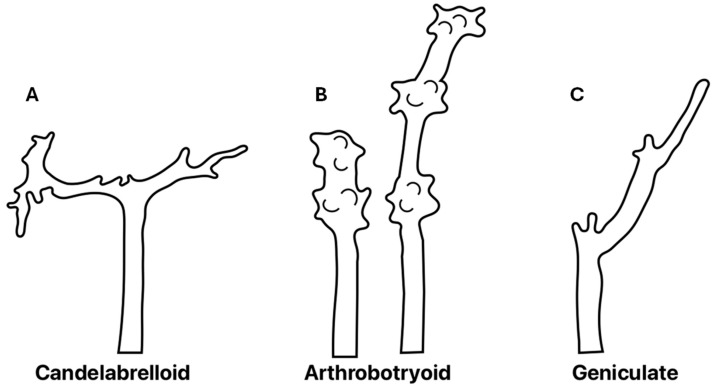
Aspects of different types of apical extremes of conidiophores of different nematophagous fungi. (**A**) The conidiophore apex bifurcates and it shows an elk horn appearance, where conidiogenesis is produced; (**B**) The conidiophore keeps its erect position and groups of denticles are produced along the conidiophore apex; (**C**) The conidiophore shows repeated bents where conidiogenesis occurs.

Additionally, our isolate showed an important taxonomical characteristic: the conidiophore type at the top, precisely where the conidiophore produces denticles where conidia are generated (conidiogenesis). Such denticles are typical of Arthrobotryoid conidiophores (Figure 4A), and this characteristic differs from those of other species such as *A. musiformis* and *A. javanica* that, instead of these denticles, form a candelabrum where conidia are produced. The presence of conidiophores with denticle formation is typical of the species *A. oligospora* (Figure 4B). Other fungi produce conidiophores very differently, like the geniculate conidiophores shown in Figure 4C. It is important to mention that our isolate showed the presence of chlamydospores, as well as the formation of three-dimensional adhesive nets as trapping devices. These characteristics led us to classify our isolate as *A. oligospora* based on morphological analysis. Regarding the other fungal isolate recorded as AspG, this isolate showed a conidia cluster different to that of *A. oligospora*. Instead, this fungus showed the presence of 4–8 obovoidal, elongated, slightly curved and slightly constricted conidia. There was a septum slightly situated below the middle of the conidia. Elongated conidia were arranged laterally or slightly above the conidiophores. This isolate also showed the presence of the same trapping devices as *A. oligospora*, three-dimensional adhesive nets. This fungus also showed the presence of chlamydospores but possessed candelabrelloid conidiophores typical of *A. musiformis*. This structure resembles an elk horn where conidia are developed (Figure 4B). 

### 3.2. Molecular Identification

After analysing the DNA sequences of the AspT strain and comparing this information with the sequences of isolates previously reported in the NCBI database, high coverage (96–99%) and similarity (98.86–99.54%) values with respect to fungi from the species *Orbilia oligospora* and *A. oligospora* were found. On the one hand, both species seem to be synonyms according to recent nomenclature reported in the NCBI database [25] (Taxonomy ID: 2813651, NCBI:txis2813651). On the other hand, *A. musiformis* matched those sequences previously reported in the NCBI database with high levels of coverage (99.32%) corresponding to *A. musiformis* (MH855842.1., KP859624.1, OL454931.1) and *A. eryuanensis* (MT612105.1). However, there are some remarkable morphological differences between *A. musiformis* and *A. eryuanensis*. For example, the length of erect conidiophores in *A. musiformis* can reach up to 900 μM [18]; meanwhile, *A. eryuanensis* reaches a length range of 110–308 µm, and this species also produces macro- and microconidia [19]. It is important to mention that both isolates were recorded at the NCBI as *Arthrobotrys oligospora* strain INIFAP-SCv-01 (PP567275) and *Arthrobotrys musiformis* strain INIFAP-SCv-02 (PP567276). 

**Figure 5:**
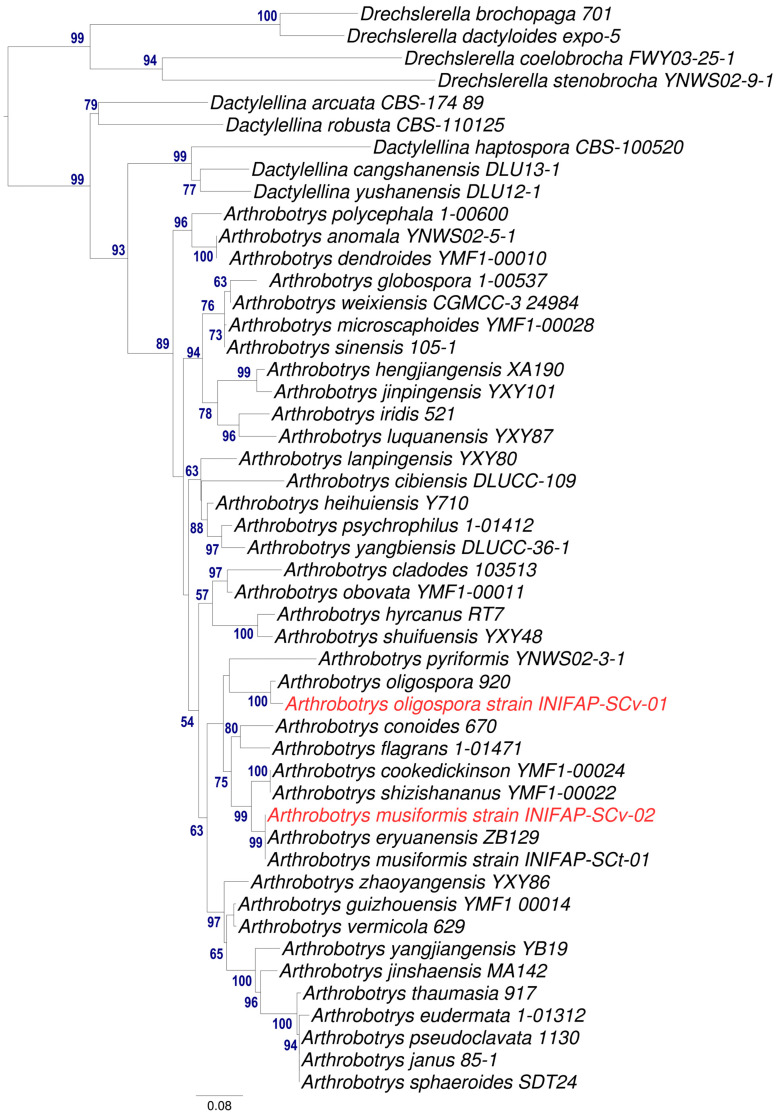
Phylogenetic analysis from the AspG and AspT fungal isolates and related species using 50 sequences from the ITS1, 2 and 5.8S region. Support values > 50% for Maximum Likelihood, are shown in nodes (in blue).

To obtain a more robust molecular taxonomic identification, a Maximum Likelyhood phylogenetic tree using ITS1,2 and 5.8S regions was created (Figure 5). This analysis revealed that our isolate AspT is closely related to *A. oligospora* (strain 920) with high BS in this node. Meanwhile, the AspG strain is closely related with two reported species *A. musiformis* and *A. eryuanensis* and the analysis did not provide sufficient evidence to differentiate our isolate from both species. To elucidate this doubt, we re-revised the morphological descriptions for both species and found important morphological differences between them. For example, *A. musiformis* forms only macroconidia, while *A. eryuanensis* produces macro and microconidia; additionally, *A. musiformis* produces erect and not branched conidiophores, opposite to *A. eryuanensis*, which produces branched conidiophores [19].

### 3.3. Predatory Activity Assessment of the Two Nematophagous Fungal Isolates against Haemonchus contortus Infective Larvae

The mean numbers of *H. contortus* infective larvae recovered from agar plate groups with and without fungi, as well as the larval reduction attributed to the predatory effect of both fungi, are shown in Table 3. *Arthrobotrys oligospora* (AspT) showed a moderate larval reduction (around 45%) after 10 days of interaction; meanwhile, *A. musiformis* (AspG) reached a higher larval reduction (>70%) (*p* < 0.05).

In the literature consulted, we found several reports where different strains of both species obtained from different sources showed variable results in terms of their predatory activity against ruminant parasitic nematodes, i.e., in a recent study, an *A. oligospora* isolate obtained from a fig plantation soil sample from Tepalcingo, Morelos, Mexico showed 73.6% predatory activity against the *H. contortus* infective larvae [26]. Likewise, Cao et al. (2018) [27], using Chinese isolates of *A. musiformis*, reported a range of 89.02% to 94.80% reduction of infective larvae of the nematode *Trichostrongylus colubriformis* [27]. In another study, an *A. musiformis* strain isolated from a soil sample from Cuautla municipality, Morelos state, Mexico showed 71.54% larval mortality using *H. contortus* (L_3_) as a target [22]. 

Other isolates of *A. oligospora* and *A. musiformis* showing variable results regarding their predatory capability against infective larvae of ruminant parasitic nematodes and other nematode targets are shown in Table 4. In this regard, we can build the following reflection: the biological behaviour of nematophagous fungi is a dynamic process and it depends on the adaptation to environmental circumstances of each microorganism from the soil microbiota; such variation in the ability to form traps and capture nematodes of different taxonomic groups can be attributed to the influence of diverse abiotic and biotic factors in fungal habitats [28].

### 3.4. Microscopic Findings about Predatory Activity of Fungi

**Figure 6:**
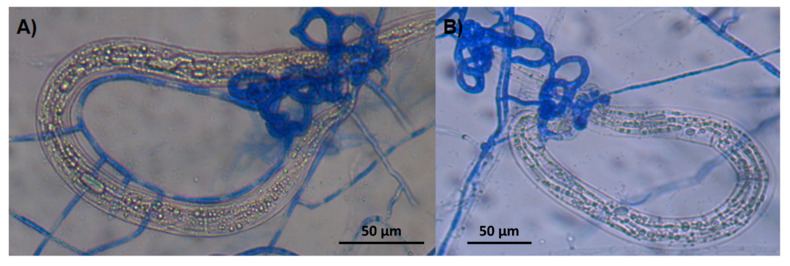
Aspect of *Haemonchus contortus* infective larvae captured in three-dimensional adhesive nets of two nematophagous fungi, *Arthrobotrys oligospora* (**A**) and *A. musiformis* (**B**).

The microscopical analysis of the agar plate surfaces of both fungi interacting with *H. contortus* larvae revealed the formation of three-dimensional adhesive nets where nematodes appeared trapped (Figure 6A, *A. oligospora* and Figure 6B *A. musiformis*). 

Both species of nematode-trapping fungi are saprophytic organisms living in soil, decaying wood, plant roots or litter and they take their requirements of nitrogen and carbon from these organic materials; however, when nematodes are in their proximity some protein products peeled from the nematode cuticle can stimulate genes responsible of regulating the morphogenesis process. This process is a physiological mechanism of fungi where they modify their mycelia and transform them into trapping devices [33]. The interaction fungi/nematodes process includes various steps, i.e., the formation of an infecting bulb (or appressoria) that exerts a physical pressure [7], and together with the production of cuticle-degrading enzymes, can penetrate the nematode body to eventually invade and take their nutritional requirements from the internal nematode tissues [34]. In both images (Figure 4), in addition to the trap formations, invasive hyphae can be seen filling the inside of the larval corps.

### 3.5. Assessment of the In Vitro Nematocidal Activity of Liquid Culture Filtrates of Arthrobotrys oligospora and Arthrobotrys musiformis Produced in SPDB and CzDB against Haemonchus contortus Infective Larvae

The results regarding the larval mortality of *H. contortus* attributed to the effect of LCF of both nematophagous fungi either individually or in combination (50:50) grown in the two evaluated media are shown in Table 5. The larval mortality caused by LCF of each fungus in individual use in both media was observed under a concentration-dependent effect; where the highest larval mortality was observed at the highest concentration used (100 mg/mL). It is important to remark that relevant differences between the individual and combined use of both fungi were not recorded. In both cases, the two LCF of both fungi showed a high lethal effect against the *H. contortus* infective larvae at the highest concentration. However, a higher larval mortality was observed in LCF obtained from CzDB in comparison with LCF obtained from SPDB. In this regard, we expected to observe a synergistic effect of the combined use of LCF; however, this effect was only found in the LCF concentration of 25 mg/mL in CzDB. No antagonistic effect in the combined use of LCF in both media was observed. In a study, the effect of LCF on different nematode-trapping fungi including *Arthrobotrys conoides*, *A. cookedickinson*, *A. eudermata*, *A. microscaphoides*, *A. oligospora*, *A. thaumasia* and *Clonostachys rosea* in combination with *Trichoderma harzianum* and the bacterium *Pseudomonas fluorescence* were assessed against eggs and juveniles of the phytoparasitic nematode *Meloidogyne* spp. and authors reported a range of juvenile mortality (J2) between 47.22–59.72% (used individually); meanwhile, the LCF combinations ranged between 65.26–73.61%; the highest juvenile mortality was achieved with *P. fluorescence* and *T. harzianum* [35]. In another study, the combined effect of the bacterium *Pseudomonas fluorescence* and the entomopathogenic fungus *Metharhizium anisopliae* was assessed against eggs and juveniles (J2) of *M. incognita*; authors reported an improvement in the nematocidal effect of the combined use of both filtrates of bacteria + fungus that reached 96.98% juvenile mortality and 98.22% egg-hatching inhibition [36]. 

In an extensive revision of the available literature, the authors did not find more “ad hoc” studies to compare with our results.

### 3.6. Microscopic Findings Regarding Haemonchus contortus Infective Larvae Exposed to Liquid Culture Filtrates of Two Nematophagous Fungi Grown in Sweet Potato Dextrose Broth and Czapek-Dox Broth

**Figure 7:**
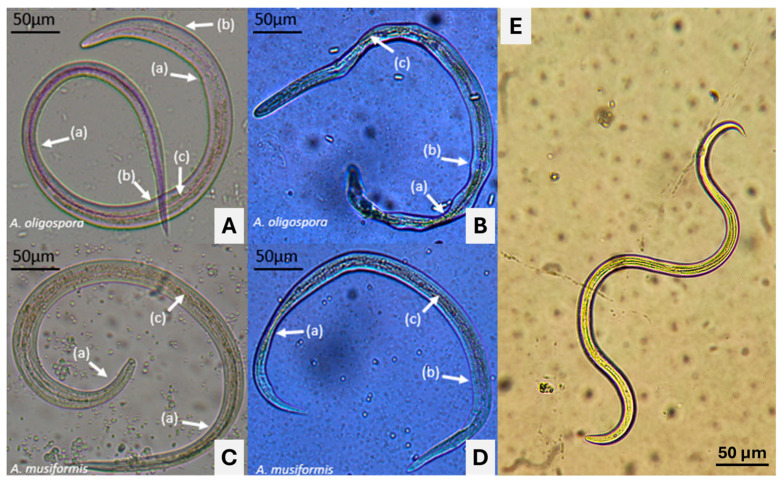
Microphotographs showing the aspect of *Haemonchus contortus* infective larvae after 72 h exposure to liquid culture filtrates obtained with two nematophagous fungi *Arthrobotrys musiformis* and *A. oligospora* grown in Czapek-Dox Broth (**A**) and (**C**) and Sweet Potato Dextrose Broth (**B**) and (**D**), and a live larva from the control group, without fungus culture (**E**). (a) deformation of the larval bodies; (b), loss of turgor body; (c), loss of the intestinal cellular structural architecture.

A set of microphotographs showing morphological changes identified in *H. contortus* infective larvae after exposure to LCF of *A. oligospora* and *A. musiformis* are shown in Figure 7. Images A and C show the aspects of *H. contortus* infective larvae after 72 h exposure to LCF of *A. oligospora* and *A. musiformis*, respectively (grown in CzDB), assessed at 100 mg/mL. In these images, a thickening of the larval bodies was observed in some areas; meanwhile, in other areas, a diminishing of the body thickness was visualized. In both cases, a clear loss of the intestinal cellular structural architecture was observed (c). Likewise, larvae exposed to LCF of both fungi grown in SPDB (B) and (D) showed similar damage, but was more severe in SPDB, and a general deformation of larval bodies with a slimming in some areas of the nematode corps (a) and loss of turgor body in other areas (b) were observed. After analysing the images captured by microscopy, we observed malformations in different sites of the larval bodies exposed to both LCFs. The larvae exposed to LCF from both fungi obtained from CzDB showed some morphological changes; however, the damage to those larvae exposed to LCF obtained from both fungi in SPBD showed more severe morphological alterations. These findings suggest that the culture media can influence the production of bioactive mycocompounds derived from the secondary metabolism of fungi. In future studies, we plan to use confocal laser scanning microscopy to perform a co-localization analysis to determine the site of action and the damage caused by metabolites responsible of the nematocidal activity [37].

### 3.7. Myco-Chemical Compound Profile

The results of the myco-quantitative reagent analysis obtained with LCF of both fungi in both culture media are shown in Table 6. The groups of secondary metabolites found after mycochemical compound analysis of LCF obtained in both liquid media (CzDB and SPDB) of the two nematophagous fungi (*A*. *musiformis* and *A*. *oligospora*) were alkaloids with a light reaction in the four treatments: coumarins with a light reaction in *A. musiformis* in SPDB and *A. oligospora* in both media. Regarding the presence of sterols, this group of compounds was found with a light reaction in LCF of *A. musiformis* in SPDB and *A. oligospora* in CzDB; however, in *A. oligospora*, a positive reaction was found in SPDB and a strong reaction in CzDB. These findings show that *A. oligospora*, despite not showing high predatory activity of *H. contortus* infective larvae using physical mechanisms (three-dimensional adhesive nets), in certain respects, compensates for this lack of a strong predatory activity by producing many secondary metabolites with nematocidal activity, which could contribute to the immobilization and killing of nematodes. Some groups of compounds identified in the present study in both species of *Arthrobotrys* have been found in other isolates of this genus and are associated with several biological activities. On the one hand, coumarins obtained from other sources i.e., plants and marine plants have been reported to possess important therapeutic activities, i.e., antifungal, anti-inflammatory and antimicrobial [38] and nematocidal activity [39,40]. On the other hand, some compounds belonging to the group of Alkaloids are also demonstrated to possess potent nematocidal activity against gastrointestinal parasitic nematodes [41] and against phytoparasitic nematodes [42]. Other compounds obtained in the present study including sterols and saponins have also been reported to have important nematocidal activity against nematodes of importance in agriculture [43,44].

## 4. Conclusions

In the present study, no enhancing effect in the nematocidal activity was identified after using the LCF of *A. oligospora* and *A. musiformis* in combination against *H. contortus* infective larvae. Liquid culture filtrates of both fungi exerted an important nematocidal effect at the highest concentration (100 mg/mL) when used either individually or in a combined manner. The results of the present study contribute important information about the predatory activity of these species of nematophagous fungi and additionally about the nematocidal activity of LCF produced by these fungi that could have implications for future studies exploring the use of this LCF or the metabolites responsible for nematocidal activity as potential tools of control against sheep haemonchosis. 

## Figures and Tables

**Table 1:** Experimental design to assess the lethal effect of liquid culture filtrates of *Arthrobotrys oligospora* and *A. musiformis* produced in two liquid media against *Haemonchus contortus* infective larvae (L_3_).

Treatment	Medium	Fungal Filtrate
1	Sweet Potato Dextrose Broth	*Arthrobotrys oligospora* ^(^*^)^
2		*A. musiformis* ^(^*^)^
3		*A. oligospora + A. musiformis* ^(^*^)^
4		Control (without fungus)
5	Czapek-Dox Broth	*Arthrobotrys oligospora* ^(^*^)^
6		*A. musiformis* ^(^*^)^
7		*A. oligospora + A. musiformis* ^(^*^)^
8		Control (without fungus)
Controls		
9	Phosphate Buffered Saline	Negative control
10	Ivermectin (0.5%)	Positive control

n = 4; ^(^*^)^ =The nematocidal effect was assessed at three different concentrations (25, 50 and 100 mg/mL).

**Table 2:** Main morphological characteristics observed in the two isolates of nematophagous fungi belonging to the genus *Arthrobotrys* and means of measurement of taxonomically important structures.

Characteristic	Strain 1 (AspT)	Strain 2 (AspG)
Conidium shape	Globose, slightly constricted in the septum (on occasion).	Ellipsoidal to obovoidal, slightly curved
Septum	One septum situated slightly below the half conidium	One septum situated slightly below the half conidium
Number of conidia per conidiophore	6 (4–8)	7 (3–11)
Conidia length (µm)	21.1 (19–23)	32.7 (27–40)
Conidia width (µm)	11.4 (10–12)	13 (11–17)
Conidiophores length (µm)	344 (193–429)	291 (141–403)
Identified species	*Arthrobotrys oligospora*	*Arthrobotrys musiformis*

**Table 3:** Results of the predatory activity of two nematophagous fungi *Arthrobotrys oligospora* (AspT) and *A. musiformis* (AspG) against *Haemonchus contortus* infective larvae on water agar plates.

Isolate (Species)	Recovered Larvae Control Group (Mean ± SE)	Recovered Larvae Treated Group (Mean ± SE)	Larval Reduction %
*Arthrobotrys oligospora*	192 ± 38.21	105 ± 26.42	45.14 ^a^
*Arthrobotrys musiformis*	197 ± 60.53	57 ± 30.35	70.95 ^b^

^a, b^ Different letters show statistical differences between groups.

**Table 4:** Results of the in vitro predatory activity of *Arthrobotrys musiformis* and *A. oligospora* isolates obtained from different sources and using different nematode targets.

Isolate	Source of Isolation	Nematode Target	Predatory Activity %	Author (s)
*A. oligospora*	Not available	*Meloidogyne incognita* (**)	79.6–87.5	[28]
*A. musiformis*	Mossy soil, decaying plant material (a rotten trunk) and soil containing Brahea palm roots	*H. contortus* (L3)	>97	[29]
*A. oligospora*	Faeces of water buffalo	*H. contortus* (L3)	>89	[30]
*A. oligospora*	Soil and animal faeces	*Panagrellus redivivus* (*)	57.2	[16]
*A. musiformis*	Soil sample	*H. contortus* (L3)	>74	[31]
*A. oligospora*	Soil samples	*Aphelenchoides besseyi, Bursaphelenchus xylophilus* and *Ditylenchus destructor* (**)	54.6–97.3	[32]

(*) =A Free-living nematode; (**) = Plant parasitic nematodes.

**Table 5:** Mean of *Haemonchus contortus* dead and total recovered larvae and mean larval mortality percentages after 72 h exposure to individual and combined liquid culture filtrates obtained from *Arthrobotrys musiformis* and *A. oligospora* produced in two liquid media.

Concentration (mg/mL)	Fungal Filtrate	DL/TL	Mortality %	SE	Significance *
Sweet potato dextrose broth					
0	*A. oligospora*	6/119	5.03	0.87	1
	*A. musiformis*	6/119	5.03	0.87	
	Combination	6/119	5.03	0.87	
25	*A. oligospora*	12/96	12.14	2.74	0.072
	*A. musiformis*	15/107	13.70	3.24	
	Combination	21/101	20.54	3.95	
50	*A. oligospora*	31/104	29.83	6.03	0.456
	*A. musiformis*	25/104	24.50	5.08	
	Combination	19/86	22.18	5.06	
100	*A. oligospora*	82/104	78.29	2.65	0.543
	*A. musiformis*	83/110	75.55	5.37	
	Combination	79/99	79.84	3.04	
Czapek-Dox Broth					
0	*A. oligospora*	2/80	2.12	0.38	0.588
	*A. musiformis*	2/86	2.65	0.40	
	Combination	2/80	2.12	0.38	
25	*A. oligospora*	23/86	27.34	5.75	0.011
	*A. musiformis*	22/81	27.08	3.42	
	Combination	37/84	44.07	5.75	
50	*A. oligospora*	38/89	43.03	8.84	0.916
	*A. musiformis*	44/86	51.59	5.54	
	Combination	36/75	48.29	7.96	
100	*A. oligospora*	75/87	86.17	3.39	0.308
	*A. musiformis*	83/91	91.35	3.05	
	Combination	81/87	92.55	2.47	

* Values < 0.05 shows that the contrast was significant; Coefficients: 0.5*A. musiformis + 0.5*Treatment 2 = 1*Treatment 3.

**Table 6:** Mycochemical profile obtained from liquid culture filtrates of two nematophagous fungi *Arthrobotrys musiformis* and *A. oligospora* grown in two culture media, Czapek-Dox Broth and Sweet Potato Dextrose Broth, by myco-quantitative reagent analysis.

Metabolites and Reagents	*Arthrobotrys musiformis*		*Arthrobotrys oligospora*	
	CzDB	SPDB	CCzDox	SPDB
Alkaloids	+	+	+	+
Coumarins	-	+	+	+
Flavonoids	-	-	-	-
Tannins	-	-	-	-
Triterpenes	-	-	-	-
Sterols	-	+	+	+
Saponins	-	+	+++	++

-: undetected reaction; +: slightly positive reaction; ++: positive reaction; +++; strong positive reaction.

## Data Availability

All the data available is included in the manuscript.
